# Automated Identification of Hidden Corrosion Based on the D-Sight Technique: A Case Study on a Military Helicopter

**DOI:** 10.3390/s23167131

**Published:** 2023-08-11

**Authors:** Andrzej Katunin, Piotr Synaszko, Krzysztof Dragan

**Affiliations:** 1 Department of Fundamentals of Machinery Design, Faculty of Mechanical Engineering, Silesian University of Technology, Konarskiego 18A, 44-100 Gliwice, Poland; 2 Airworthiness Division, Air Force Institute of Technology, Ks. Bolesława 6, 01-494 Warsaw, Poland; piotr.synaszko@itwl.pl (P.S.); krzysztof.dragan@itwl.pl (K.D.)

**Keywords:** D-Sight, hidden corrosion, damage identification, DAIS, non-destructive testing, aircraft structures

## Abstract

Hidden corrosion remains a significant problem during aircraft service, primarily because of difficulties in its detection and assessment. The non-destructive D-Sight testing technique is characterized by high sensitivity to this type of damage and is an effective sensing tool for qualitative assessments of hidden corrosion in aircraft structures used by numerous ground service entities. In this paper, the authors demonstrated a new approach to the automatic quantification of hidden corrosion based on image processing D-Sight images during periodic inspections. The performance of the developed processing algorithm was demonstrated based on the results of the inspection of a Mi family military helicopter. The nondimensional quantitative measurement introduced in this study confirmed the effectiveness of this evaluation of corrosion progression, which was in agreement with the results of qualitative analysis of D-Sight images made by inspectors. This allows for the automation of the inspection process and supports inspectors in evaluating the extent and progression of hidden corrosion.

## 1. Introduction

Widely applied regulations for periodic inspections of aircraft structures using non-destructive testing (NDT) techniques are one of the crucial tasks within the damage tolerance philosophy. According to this philosophy, such inspections ensure the timely detection and identification of damage and the further monitoring of its growth in defined limits, which makes it possible to guarantee appropriate reliability and, therefore, the structural integrity and safety of aircraft. The need for effective inspection methods for such structures drives the development of new NDT techniques and the enhancement of existing ones to improve their sensitivity to various types of damage, as well as the smaller and smaller sizes of damage sites that are possible to detect.

Regardless of the wide application of composite materials in aircraft structures, there are still numerous elements made of metallic alloys in both new and older aircraft, which means adjusting the inspection approaches to these elements and structures. Under the umbrella of structural aging, besides fatigue, one of the most widespread and costly types of damage in such structures is corrosion. The cost of aircraft corrosion is on the level of billions of dollars annually in the United States alone, according to various reports [1,2,3,4]. Moreover, as reported in [5], corrosion is a primary cause of structural issues (80% of all issues) related to the aging of aircraft, resulting in tens of incidents annually caused by this phenomenon. This demonstrates the need for effective inspection techniques to detect and identify corrosion in a timely manner. For this purpose, corrosion prevention and control programs that regulate inspection frequency, applied techniques, and corrosion identification, have been introduced based on the recommendations of the Federal Aviation Administration (FAA) advisory circular [6] and manufacturer recommendations for specific aircraft.

A variety of corrosion types are described in detail, e.g., in [2,6,7,8], and often require various approaches for their detection and evaluation. The most widely used approach for the inspection of corrosion in aircraft structures is visual inspection, which is used to detect corrosion spots. However, in some cases, visual inspection can be ineffective, especially in cases of so-called hidden corrosion, which appears primarily in rivet joints because of moisture penetration in lap joints and results in the initiation of electrochemical processes. These processes cause the appearance of corrosion products, which, given their much higher stiffness compared with the surrounding material—usually aluminum alloys—imply internal stresses, resulting in the appearance of surface deformations [9]. Because of this, the process is called the pillowing effect. Because hidden corrosion, even at high severity, is barely visible to the naked eye, NDT-based approaches are necessary for sensing and detection. According to the literature [2,3,6,10,11], the following NDT techniques are used most often in such inspections: X-ray radiography/tomography, ultrasonography, eddy current testing, thermography, and shearography. However, their application is often biased by a certain amount of uncertainty in detecting hidden corrosion spots because of the low magnitude of the resulting deformations and the specificity of the application of particular techniques. For example, the application of the eddy current NDT technique is difficult in such cases because of the allowable tolerances for manufactured sheets in the aviation industry, and therefore, the detected spots of hidden corrosion are on a level of measurement uncertainty, as reported by Komorowski et al. [12]. Moreover, all of the above-mentioned NDT techniques are characterized by high labor intensity, a relatively long period of testing, the need to dismantle tested elements in numerous situations, and comparatively high inspection costs since specially trained personnel are needed [6]. Considering this, effective, fast, accurate, and inexpensive techniques for inspection of this type of damage are favorable.

The technique that meets all these requirements is double-pass retroreflection, also known also by its commercial name—D-Sight—developed in 1983 by Diffracto Ltd. (Windsor, ON, Canada) in Canada for hidden corrosion detection in aircraft structures and successfully implemented in 1988 by the Canadian Institute for Aerospace Research National Research Council. D-Sight is an optical technique based on the evaluation of surface deformations. In this way, the mentioned problems of measurement uncertainties do not take place. The principle of operation for the D-Sight technique is based on imaging the tested surface at an oblique angle. This surface is illuminated with a light source shifted from the camera, and the light is reflected from the surface onto a special retroreflective screen, which disperses this light and reflects it back on the test surface. This makes it possible to highlight tiny deformations caused by the pillowing effect, and this image is then captured by the camera. The mathematical background for the principle of operation of this technique can be found, e.g., in [13]. The attempts of the inventors and the first working group that used the D-Sight technique for the evaluation of hidden corrosion can be found in [14,15,16]. The construction of the test device used to sense the hidden corrosion based on the D-Sight technique, known as the D-Sight Aircraft Inspection System (DAIS), is simple, which makes inspection comparatively inexpensive. Moreover, the inspection of large areas of aircraft is possible in a short testing time, which introduces savings in labor intensity. This makes it possible to perform inspections in a fast and reliable way. Nevertheless, in addition to the mentioned advantages, the D-Sight technique is mostly used as a qualitative technique, and it cannot provide enough information for the evaluation of the extent of corrosion or its growth. Several attempts have been made in the past to improve this technique by incorporating supporting finite element models and analysis of profiles based on grayscale images resulting from inspections [12,17,18,19]; however, their applicability under routine inspection conditions was still limited. Moreover, the authors of this paper identified additional difficulties during previous studies [20], namely that the angle of observation of a tested structure and the illumination should be strictly repeatable, which is especially important during the comparison of historical data from inspections. Additionally, the determination of corroded areas, visible as darker regions in D-Sight images, is also a challenging problem because of small differences in the colors of healthy and corroded areas, as well as the nonuniformity of color distribution in the vicinity of rivets, where the hidden corrosion appears. However, these areas are observable by the naked eye in D-Sight images; therefore, it is reasonable to apply perceptual color contrast measures. Numerous approaches have been developed for this purpose, which can be found in the literature. They include numerous contrast measures reviewed, e.g., in [21,22,23,24]. Most of them, however, use sophisticated algorithms, which can extend processing runtimes. The selection of an effective and fast algorithm to evaluate hidden corrosion remains an open question in the processing of D-Sight images.

Recently, numerous steps have been taken toward improving the D-Sight technique to become a quantitative system. Brandoli et al. [25] demonstrated the application of deep neural networks (DNNs) for the detection of hidden corrosion in aircraft fuselage structures. A similar approach was presented by Zuchniak et al. [26], where the authors used machine learning to detect hidden corrosion spots. Nevertheless, the problem of the quantitative evaluation of hidden corrosion remains of great importance from the point of view of supporting inspections and ground maintenance for aircraft. Some steps toward solving the mentioned disadvantages of D-Sight inspections and the quantification of hidden corrosion based on D-Sight images were undertaken by a team of authors in the following study. In [27], the authors proposed a method of image processing that includes procedures to reduce the influence of the angle of observation and non-uniform illumination and detect corroded areas, and it included the first attempts at their quantification. Furthermore, laboratory tests were performed on specimens with simulated hidden corrosion to evaluate the sensitivity of the D-Sight technique and find a correlation between the true dimensions of the corrosion spots obtained using reference methods, both planar and in the direction normal to the surface of the tested structures, as well as those estimated based on image processing of D-Sight images [28].

The current study is motivated by the need to develop a computationally efficient method for quantifying hidden corrosion in aircraft structures, which will allow for the automation of the evaluation process and support inspectors in the evaluation of the extent of corrosion and its growth over the years of an aircraft’s operation. Such evaluation is possible using subsequent analyses of D-Sight images collected during periodic inspections using this technique. The D-Sight technique is used as a routine approach for inspections at the Air Force Institute of Technology in Warsaw, which performs maintenance on aircraft for the Polish Armed Forces. The study was carried out on the inspection results of selected structures of the Mi family military helicopters to demonstrate the performance of the proposed approach using realistic inspection results. It highlights the difficulties and open questions in the process of evaluating hidden corrosion and demonstrates the processing algorithm, which can be used as a supporting tool for inspectors using this technique.

## 2. Inspections and Data Acquisition

### 2.1. Pillowing Phenomenon and Challenges of D-Sight Inspections

**Figure 1:**
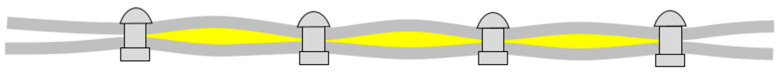
Schematic representation of pillowing phenomenon in multilayered lap joints.

In practice, hidden corrosion occurs most often in the multilayer lap joints of aluminum skins joined by rivets. The increase in the volume of aluminum oxides is greater than the decrease in volume caused by the loss of layer thickness, which results in growing deformations in the skin between the rivets (see Figure 1).

The observation of deformations allows us to detect corrosion, which may also occur in the second and subsequent layers of the lap joint. The stiffness of the aluminum oxides is larger than that of the aluminum alloys, which leads to skin deformation as well as layer thickness decreases. In this process, trihydrate oxides have a dominant influence on the resulting deformations, which is additionally amplified by the appearance of monohydrate oxides [12]. An increase in the volume of the oxides leads to not only material loss but also to pillowing deflection in the skin, which also increases the shear stress in the lap joints. This may lead to critical failures, such as multiple-site fatigue cracking, which, for example, was the main cause of cracked elements that led to failure in the infamous Aloha Airline Flight 243 accident in 1988 [29].

As discussed above, because of the small magnitude of deformation resulting from the pillowing effect, hidden corrosion is difficult to detect with numerous NDT techniques, especially in the early stages of its development. The results of previous studies [28] have shown that the lowest detectable magnitude is at the level of 30 μm. This result was obtained using the D-Sight NDT technique, which demonstrated a high sensitivity to such deformations.

**Figure 2:**
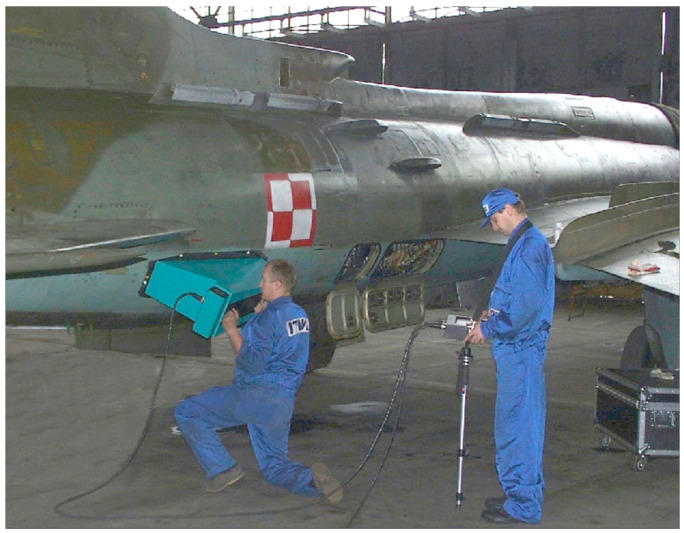
The inspection of an aircraft using DAIS.

Inspections using D-Sight techniques have been implemented with hardware created by Diffracto Ltd. in a system known as DAIS. The principle of operation for this testing device is based on the above overview of the D-Sight testing approach and can be found in numerous previous publications; see, e.g., [27,28]. However, during inspections, numerous factors influencing the quality of the resulting D-Sight images need to be taken into consideration, such as the reflectivity of the tested surface and the position of the testing device, which has a direct influence on the angle of observation and illumination. Considering the curvatures present in aircraft fuselage structures, ensuring these conditions is not always a trivial task, as can be seen in the example photograph from the inspection (see Figure 2). Because of this, the D-Sight technique is currently used in the practice of aircraft inspections mostly as a qualitative approach, which allows for the evaluation of the severity of corrosion based on the subjective opinion of a single inspector.

### 2.2. Inspections and Acquisition of D-Sight Images

**Figure 3:**
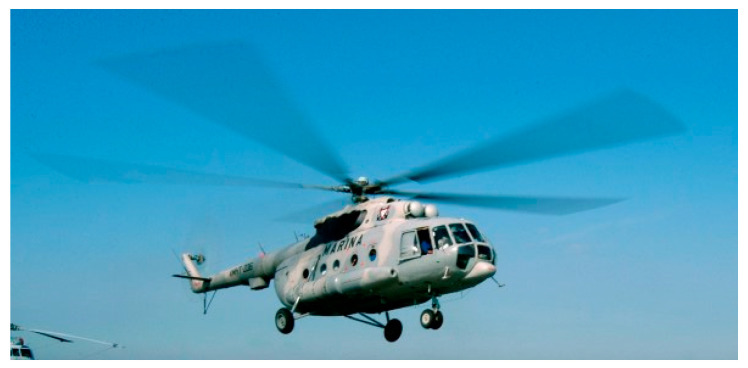
The Mi-type helicopter.

**Figure 4:**
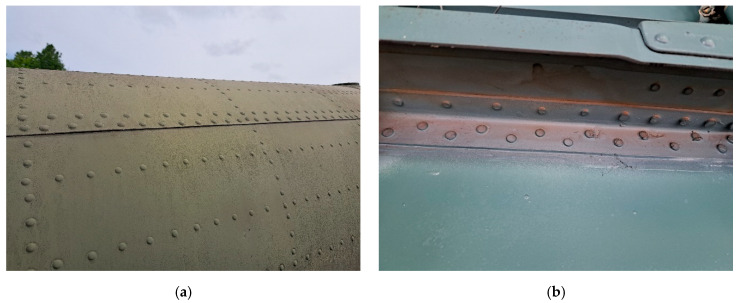
Examples of riveted lap joints in the tested helicopter: views from the outer (**a**) and inner (**b**) sides.

The current study focuses on the improvement of the D-Sight technique for the purpose of quantitatively evaluating D-Sight images and automating the evaluation process based on real aircraft structures after successfully testing the developed approach on specimens with artificially introduced deformations that simulate hidden corrosion [28]. For this purpose, historical data from periodic inspections of the Mi family of military helicopters (see Figure 3), which are a part of the arsenal of the Polish Armed Forces, were considered as a case study. According to FAA, European, and national recommendations, inspection data should be collected and analyzed throughout the service life of helicopters, especially for riveted lap joints. Some examples of the tested structures are presented in Figure 4.

**Figure 5:**
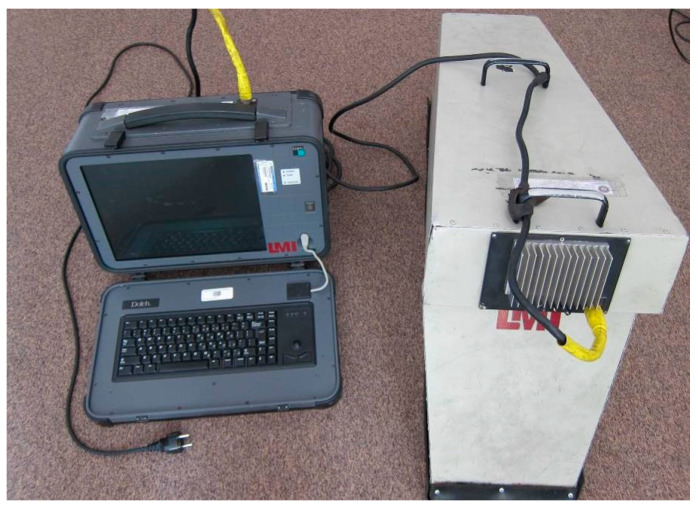
The DAIS 250C scanning system.

The inspections were carried out by the Air Force Institute of Technology, Warsaw, using the DAIS 250C scanning system (Diffracto Ltd., Windsor, ON, USA) (Figure 5).

**Figure 6:**
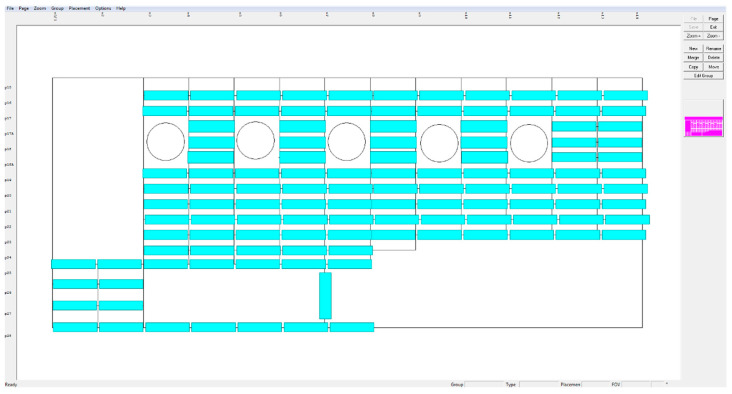
Scheme of the grid for the tested helicopter, indicating the location of interest.

Before testing, the surface is covered with Ecolink Electron^®^ antireflective agent (Tucker, GA, USA) to maximize light reflection. The role of the hood, visible in Figure 5, is to isolate the measurement system from ambient light. Images with spatial resolutions of 640 × 480 pixels are collected during inspections in accordance with a scheme of a given element, which is recoded in the settings file in the DAIS system. An example of such a schematic for the tested helicopter is presented in Figure 6. The cyan rectangles represent the areas marked for the expected appearance of hidden corrosion. The inspector reads information about the subsequent positions of the measuring device from the file, which allows him to conduct the measurements in an orderly manner.

**Figure 7:**
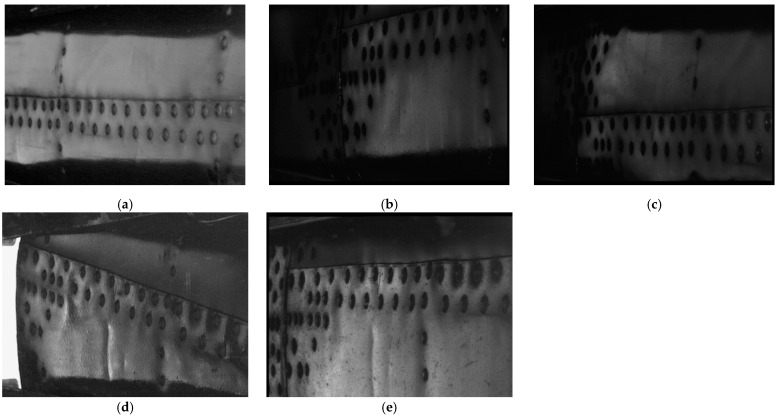
The collected D-Sight images from the inspections performed in (**a**) 2009, (**b**) 2012, (**c**) 2014, (**d**) 2017, and (**e**) 2022 for the same area of the inspected helicopter.

For the following case study, a single location of interest was selected to demonstrate the performance of the developed quantification procedure. Images acquired from the same location were collected in an inspection period of 13 years of operation for the considered helicopter. During this period, five inspections were performed. The resulting D-Sight images from these inspections are presented in Figure 7. The hidden corrosion in these images manifests in local color changes around the rivets. The corrosion severity for the tested area was classified by an inspector as small for the images collected in the period of 2009–2014 (see Figure 7a–c) and moderate for the subsequent period (see Figure 7d,e). The presented results of the inspection demonstrate the mentioned challenges in the quantitative evaluation of hidden corrosion: all of the D-Sight images have different angles of observation and illumination. Moreover, one can observe inaccuracies in the spatial positioning of the testing system, resulting in an offset observable in these images, which is a common situation in inspection practices due to the performance of inspections by various inspectors in various conditions as well as a lack of positioning systems for testing.

## 3. Processing and Evaluation of D-Sight Images

### 3.1. Data Preparation and Processing Algorithm

**Figure 8:**
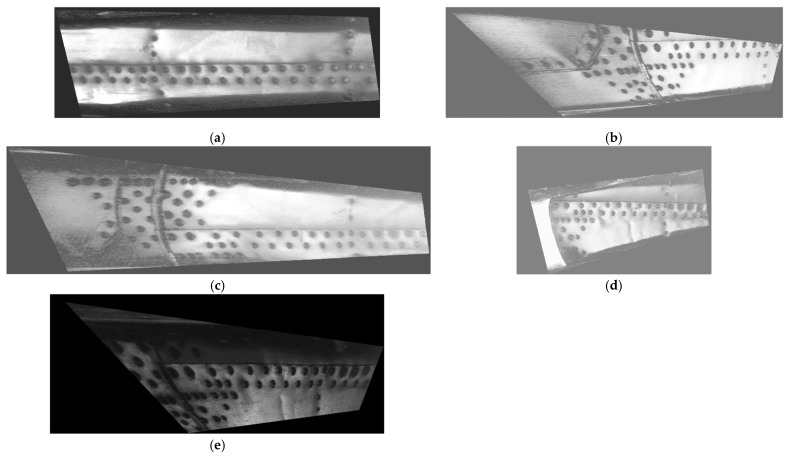
The preprocessed D-Sight images of the analyzed sequence for the tested area for the inspections performed in (**a**) 2009, (**b**) 2012, (**c**) 2014, (**d**) 2017, and (**e**) 2022.

To evaluate the extent of corrosion in the tested area and its evolution, the acquired D-Sight images required preprocessing to rotate them to a planar view. The algorithms described below were implemented using the MATLAB 2022a (MathWorks^®^, Natick, MA, USA) environment with the Image Processing Toolbox. The calculations were performed on a Windows 10 laptop equipped with an Intel^®^ Core™ i7 quad-core processor and 16 GB of RAM. The preprocessing algorithm, developed previously and in [28], consists of three main steps: image alignment, orthonormalization, and illumination equalization. The parameters of the algorithm can be found in [28]. In the first step, the edge detection procedure was applied, and then, the images were subjected to the application of Hough and shearing transforms. In the next step, orthonormalization was performed to rotate the images to a planar view, which allows for the evaluation of the dimensions of the corrosion spots. In the last step, the contrast of the images was improved to highlight the color differences in the hidden corrosion spots in the vicinity of rivets. The preprocessed D-Sight images of the analyzed sequence are presented in Figure 8. The color difference in the vicinity of rivets, which represents hidden corrosion spots, is still well visible in the preprocessed images.

Based on an analysis of the literature, the local ΔE* metric was selected to evaluate perceptual color differences. To detect and quantify the corrosion spots, the preprocessed images were converted into the CIELAB color space, known also as the L*a*b* color space, according to the CIE76 standard proposed by the International Commission of Illumination (Commission internationale de l’éclairage) in 1976. The conversion into this color space is due to its perceptual uniformity within the human eye, which makes it possible to measure color differences. Next, the ΔE* metric, defined as

(1) $$∆E^{*}=\sqrt{{\left(L_{2}^{*}-L_{1}^{*}\right)}^{2}+{\left(a_{2}^{*}-a_{1}^{*}\right)}^{2}+{\left(b_{2}^{*}-b_{1}^{*}\right)}^{2}}$$

where $\left(L_{1}^{*},a_{1}^{*},b_{1}^{*}\right)$ and $\left(L_{2}^{*},a_{2}^{*},b_{2}^{*}\right)$ represent two colors defined in the L*a*b* color space, was applied to converted images to quantify color differences. This metric is based on the calculation of the Euclidean distance in particular channels of the L*a*b* color space. To limit this approach to local color changes, the square window with a 20 times smaller size with respect to the height of the analyzed image was applied. This value was determined empirically and adjusted to typical dimensions of the corrosion spots in the analyzed images. The introduction of such a dependency allowed for a reduction in differences in the dimensions of the images obtained after preprocessing (Figure 8). Using this window, the local mean values $\left({\bar{L}}^{*},{\bar{a}}^{*},{\bar{b}}^{*}\right)$ in each channel of the L*a*b* color space were calculated, and then, the original values of the analyzed image in the specified window were subtracted from this local mean value. In this way, the terms of the Euclidean distance formula were obtained, being a modification of (1):

(2) $$∆E^{*}=\sqrt{{\left({\bar{L}}^{*}-L_{1}^{*}\right)}^{2}+{\left({\bar{a}}^{*}-a_{1}^{*}\right)}^{2}+{\left({\bar{b}}^{*}-b_{1}^{*}\right)}^{2}}.$$

**Figure 9:**
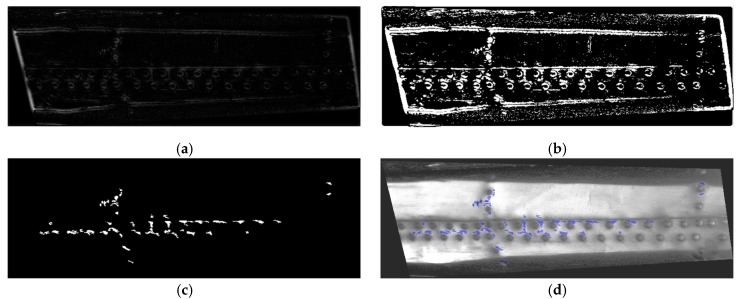
The results of subsequent operations during the processing of the preprocessed D-Sight images: (**a**) determination of the local ΔE* metric; (**b**) quantization; (**c**) morphological operations; (**d**) visualization of the detected corrosion spots.

The results presented in [21] show that the applied approach is the fastest among similar and more advanced algorithms. The example of an image obtained after these operations for the case shown in Figure 8a is presented in Figure 9a. Then, a quantization procedure was performed on an analyzed image using thresholding based on Otsu’s method. The thresholds determined during this procedure were adjusted to the color intensity of an analyzed image, which additionally reduced the problem of non-uniform illumination within the sequence of D-Sight images being analyzed. The result of this procedure is presented in Figure 9b.

**Figure 10:**
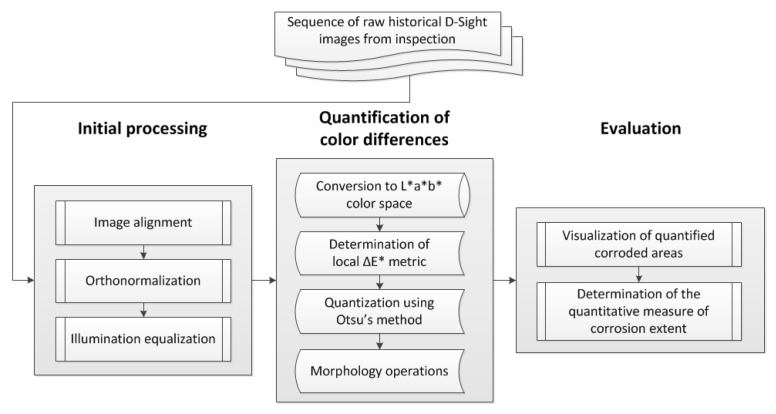
The flowchart of the processing algorithm.

Further, to identify corroded areas and rivets, morphology operations were applied. In the beginning, very large and very small objects that correspond to the surrounding frames of the tested structures and the long edges of overlapping sheets, and noise, respectively, were removed using a morphological opening. The threshold for very large objects was set to 100,000 px, while the threshold for very small objects was calculated as the total area of an image in px divided by 12,000; these thresholds were selected empirically by analyzing the considered images and were further applied within XOR logical operation. In the next step, to classify corrosion spots and rivets, the following criteria were applied. The rivets were classified based on the criterion of the roundness of the convex areas of the remaining objects, while the corrosion spots were classified based on the aspect ratio of the bounding boxes of the remaining objects with a threshold set at five. All objects for which this threshold was exceeded were removed from the image. The latter operation allowed for the removal of residues from the surrounding frames and shadows inappropriately classified as corrosion spots after the initial cleaning. The resulting image after the application of morphological operations is presented in Figure 9c. In the last step, the corrosion spots were visualized as a mask on the preprocessed D-Sight image (see Figure 9d), and the nondimensional corrosion extent was calculated using the following quantitative measure. To determine its value, the determined areas of the convex areas of the detected rivets (the number of considered rivets was in a range of 20–40) were averaged and then used as a divisor for the total area of the detected corrosion spots. Given the size of the D-Sight images after preprocessing, the runtimes for each case were ca. 30 min. For clarity, the processing algorithm is presented in the form of a flowchart in Figure 10.

### 3.2. Evaluation of Hidden Corrosion

**Figure 11:**
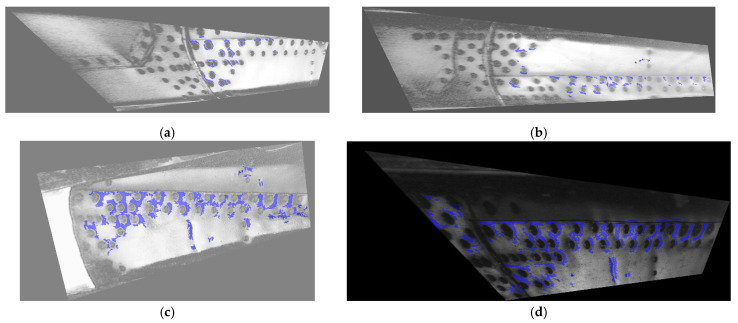
The results of processing the preprocessed D-Sight images based on the analyzed historical sequence, which were captured in (**a**) 2012, (**b**) 2014, (**c**) 2017, and (**d**) 2022.

The sequence of D-Sight images analyzed in this study (see Figure 7) was processed using the algorithm presented in Section 3.1. The results of processing are presented in Figure 9d and Figure 11a–d.

From the presented sequence, the progression of the hidden corrosion that developed over the tested helicopter’s years of operation is clearly visible; i.e., the corroded area around the rivets, labeled with a blue mask in the preprocessed D-Sight images, significantly increased. It can be seen that, in some locations, hidden corrosion was not properly detected (see, e.g., the vertical line of rivets on the bottom right in Figure 11d); however, the vast majority of the corrosion spots were well detected and identified. The corroded areas were also assessed quantitatively based on the measure introduced in Section 3.1. The selection of this measure was based on the simplicity of its implementation and runtime efficiency, which is a critical parameter during the automated evaluation of D-Sight images. The obtained results are presented in Table 1.

The determined quantitative measure of the analyzed sequence of D-Sight images demonstrates the increasing trend of the extent of the corrosion, which is in agreement with the results of the qualitative assessment of corrosion severity made by the inspector (see Section 2.2), despite the mismatches in the observed corroded areas (the spatial offset between particular D-Sight images during subsequent inspections). These mismatches are the reason for the decrease in the value of the quantitative measure of the inspection in 2012. Nevertheless, the obtained quantitative results confirm the trend in corrosion progression in the analyzed period of operation for the tested helicopter and can be used as an automated supporting tool for quantitative assessments of the extent and progression of hidden corrosion.

## 4. Discussion and Conclusions

The presented case study demonstrates an approach to processing D-Sight images collected during periodic inspections of a selected area of a fuselage of a Mi-family military helicopter, which allows one to quantify the extent of hidden corrosion and its progression in an automatic way. The results of the case study show numerous challenges during the quantitative analysis of corroded areas (variable angle of observation, inhomogeneous and non-repeatable illumination, spatial offsets of the acquired D-Sight images), which have the precedent in the repeatable conditions of inspections, being difficult to maintain over long service periods. An improvement in the repeatability of the performed inspections may significantly improve the analysis of the acquired inspection results. As demonstrated in this paper, this is especially important for quantitative evaluations of corrosion extent and progression. Despite the mentioned challenges, the presented processing algorithm, which is based on the determination of the local ΔE* metric, demonstrates high sensitivity to changes in colors in the vicinity of rivets and allows for the successful identification of corroded areas. The obtained quantitative results for the analyzed sequence of D-Sight images demonstrated the increasing trend in the total area of the corrosion spots and are in agreement with the results of the qualitative analysis performed by the inspector, despite the aforementioned challenges and uncertainties, which consist of, among others, spatial offset from inspection to inspection. To further automatize the process of tracking the hidden corrosion growth, the problem of this offset needs to be solved, which is currently one of the limitations of the algorithm and is planned to be resolved during further studies. Moreover, currently the algorithm requires a lot of time to process D-Sight images, mainly due to the significant extension of the size of images after the pre-processing step (see Figure 10). From the point of view of further analysis, such resolution seems to be unnecessary; therefore, optimal scaling needs to be applied in further steps to reduce runtimes and retain the detectability of corrosion spots. Finally, as observed in Figure 11c,d, some dents present on the surface of the tested element were classified as hidden corrosion, since the deformations were similar to those of the appearance of hidden corrosion. This requires checking the results by an inspector to detect such cases, which is another limitation on the way of automating of this approach to be solved in future studies.

The automation of the damage extent and severity assessment process, based on the processing of acquired D-Sight images, makes it possible to effectively support inspectors in the evaluation of the extent, severity, and progression of hidden corrosion during service periods. Moreover, the possibility of quantitative analysis opens up new perspectives that were not available when the technique had a qualitative character, e.g., the possibility of predicting hidden corrosion progression and the development of maintenance programs. Such an approach may influence cost reductions in maintenance and increase the availability of helicopters. This requires further deep studies, which will consolidate knowledge about electrochemical processes during the appearance of this type of corrosion with the obtained results in the following and preceding studies; analyses of loading and environmental factors, which also have an influence on the process; and numerous other factors. The development of such models should be considered in future authors’ studies. Finally, the automatic evaluation of the extent of corrosion using D-Sight images makes it possible to prepare large datasets that can be used to train DNNs to further improve the effectiveness of the identification of hidden corrosion spots and avoid their incorrect identification in the case of the presence of other deformed areas.

## Figures and Tables

**Table 1:** The results of the quantitative analysis of corroded areas for the analyzed sequence of D-Sight images.

Year of Inspection	2009	2012	2014	2017	2022
Value of quantitative measure	13.1842	12.6146	14.0894	39.7338	52.5461

## Data Availability

The data that support the findings of this study were taken from the Air Force Institute of Technology. Restrictions apply to the availability of these data, which were used with permission for this study.
